# Evaluation of gastrointestinal tract lesions and serum malondialdehyde levels after repeated oral administration of phenylbutazone in horses

**DOI:** 10.1007/s11259-024-10415-y

**Published:** 2024-05-21

**Authors:** Parichart Tesena, Ruethaiwan Vinijkumthorn, Titirat Preuksathaporn, Poonnada Piyakul, Thanapon Chotikaprakal, Rannaree Sirireugwipas, Kanokpich Wong-aree, Nawarus Prapaiwan

**Affiliations:** 1 Department of Clinical Sciences and Public Health, Faculty of Veterinary Science, Mahidol University, Nakhon Pathom, 73170 Thailand; 2 Faculty of Veterinary Science, Mahidol University, Nakhon Pathom, 73170 Thailand; 3 Veterinary and Remount Department, Royal Thai Army Animal Hospital, Nakhon Pathom, 73000 Thailand

**Keywords:** Abdominal ultrasound, Equine, Equine gastric ulcer syndrome, Gastroscopy, NSAIDs, Oxidative stress

## Abstract

Phenylbutazone (PBZ) is a widely used nonsteroidal anti-inflammatory drug for horses. However, because of its gastrointestinal side effects, its administration requires careful attention in veterinary practice. Malondialdehyde (MDA) is a serum biomarker associated with increased damage to the equine gastrointestinal system. This study investigated the hematological effects and alterations in the gastrointestinal tract and assessed serum MDA concentrations following repeated oral PBZ administration at clinical doses. Fourteen horses were randomly divided into control and treatment groups. All horses in the treatment group were administered 4.4 milligrams per kilogram of body weight of PBZ syrup orally twice a day for 7 days, whereas the control group received syrup as a placebo. The development of gastrointestinal side effects was investigated using gastroscopy, abdominal ultrasound, and fecal pH; serum MDA concentrations were assessed using a commercially available enzyme-linked immunosorbent assay kit. Data were compared between PBZ-treated and control horses before and after the treatment period. The treatment group exhibited decreased albumin and total protein concentrations. Moreover, this group exhibited a higher thickness of the right dorsal colon wall (*p* = 0.03) and had higher scores for squamous gastric ulcers (*p* = 0.01). Fecal pH was lower in the treatment group than in the control group after PBZ administration (*p* < 0.01). Although MDA concentrations were higher in the treatment group after PBZ administration, they did not differ significantly from those of the control group. This study highlighted the changes in hematological and gastrointestinal lesions resulting from PBZ administration in horses at clinical doses, even without clinical signs. However, MDA may not be an optimal biomarker for the early detection of gastrointestinal damage due to PBZ treatment in horses.

## Introduction

Phenylbutazone (PBZ) is one of the oldest nonsteroidal anti-inflammatory drugs (NSAIDs) used in veterinary medicine, second only to aspirin (Tobin et al. 1986). It is still commonly used in horses for treating pain and inflammation caused by musculoskeletal disorders and soft tissue injuries (Jacobs et al. 2022; Soma et al. 2012). The main effect of NSAIDs is the inhibition of cyclooxygenases (COXs), which convert polyunsaturated fatty acids into prostaglandins (PGs) during the inflammatory process (Blobaum and Marnett 2007). Unfortunately, NSAIDs can cause adverse effects in both humans and animals, especially in the gastrointestinal tract. In horses, NSAID administration can lead to various gastrointestinal adverse effects. Many Studies suggest that NSAIDs can contribute to the development of Equine Gastric Ulcer Syndrome (EGUS), in particular to Equine Gastric Glandular Disease (EGGD) (Pedersen et al. 2018; Richardson et al. 2018). Additionally, NSAID use is associated with the development of NSAID-induced enteropathy, a type of protein-losing enteropathy that often affects the right dorsal colon (RDC), thus is commonly known also as right dorsal colitis (Cohen et al. 1995; Richardson et al. 2018). Right dorsal colitis is localized in the RDC and can be caused by the use of NSAIDs (Davis 2017). When given at the recommended dose and dosing interval, horses usually tolerate PBZ (Goodrich and Nixon 2006). However, individual sensitivity to NSAID toxicity can lead to potential side effects even at the recommended doses (Flood and Stewart 2022). Therefore, veterinarians must be able to identify early symptoms, perform diagnostic procedures for NSAID toxicosis, and adopt a multimodal approach to analgesia whenever possible (Cook and Blikslager 2015). Currently, the gold standard for diagnosing EGUS is gastroscopy (Shawaf et al. 2020), whereas ultrasound evaluation of RDC wall thickness helps diagnosing right dorsal colitis (van Galen et al. 2021). Nonetheless, further research would be beneficial to explore and develop methods that are less invasive and allow earlier detection of NSAID-induced gastrointestinal side effects. Proteomics can be a helpful tool in early diagnosis of various diseases, including EGUS. Ten protein markers for equine squamous gastric disease (ESGD) and 14 for EGGD have been successfully identified through proteomics (Tesena et al. 2019). Some of these peptides are biomarkers of oxidative stress, and oxidative stress seems to play an important role in various equine gastrointestinal diseases, including colic (Hajimohammadi et al. 2023), EGGD (Banse and Andrews 2019), EGUS (Shawaf et al. 2020), and colitis (El-Ashker et al. 2012), by causing injury to the gastrointestinal mucosa. Thus, the measurement of oxidative stress biomarkers could be an option for less invasive diagnostic evaluation. However, it is unknown whether any of these biomarkers would be helpful in performing early diagnosis of NSAID-induced gastrointestinal damage, before onset of clinical signs. Malondialdehyde (MDA) is an established biological marker of cell membrane lipid peroxidation induced by oxidative stress. It is a major metabolite of arachidonic acid and is recognized as a reliable biomarker of oxidative stress (Singh et al. 2014). Among serum oxidative stress biomarkers, MDA has been identified as an indicator of NSAID-induced lesions in the gastrointestinal tract of experimental animals (Demircan et al. 2005; Fornai et al. 2016) and horses (Naito et al. 1998). In horses, an increase in the average plasma MDA concentration was observed 96 h after oral PBZ administration (Zuluaga Cabrera et al. 2016). Moreover, serum MDA concentrations were significantly higher in horses with EGUS under field conditions than in clinically healthy horses, indicating that MDA could be used as a preliminary screening marker for EGUS (Shawaf et al. 2020).

Furthermore, MDA was significantly associated with increased mortality among draft horses with PBZ-induced colitis under field conditions (El-Ashker et al. 2012). In contrast, no significant difference was observed in MDA levels on RDC tissue between the control group and horses with right dorsal colitis treated with prolonged PBZ administration (8.8 mg/kg body weight orally once daily for 21 days) (McConnico et al. 2008). However, to the best of the authors’ knowledge, the possible effects of short-term oral PBZ administration at a clinical dose on MDA plasma concentrations and their associations with the development of gastrointestinal lesions have not been investigated yet. This information could be valuable for monitoring horses undergoing clinical treatment with PBZ, with the goal of early detecting potential gastrointestinal complications that could pose life-threatening risks if not promptly addressed. The first objective of this study was to assess hematological and gastrointestinal changes in horses receiving a short course treatment with PBZ at clinical doses. We hypothesized that at clinical doses for a short period, PBZ would induce subclinical hematological and gastrointestinal changes, but not obvious clinical disease. The second objective was to explore the potential utility of MDA as a biomarker for early detection of PBZ-induced gastrointestinal side effects in horses. We hypothesized that after short-term PBZ treatment, a significant increase in MDA plasma concentration would be observed in parallel with subclinical hematological and gastrointestinal changes.

## Materials and methods

### Animals

Fourteen horses, including mares (*n* = 4) and geldings (*n* = 10), weighing between 366 and 532 kg and aged 4–20 years, from the Veterinary and Remount Department in Nakhon Pathom, Thailand, were enrolled in this study. Sample size was calculated using statistical power analysis software (GPower version 3.1, Dusseldorf, Germany). A priori power analysis with alpha error is used to calculate the sample size. Horses were determined to be healthy based on physical examination, history, bloodwork (complete blood count (CBC) and biochemistry), normal fecal pH, and negative fecal screening for parasites (flotation). No horse had a history of NSAID administration within the 3 weeks preceding the experiment. In all horses, prior to study inclusion, baseline gastroscopy revealed an EGUS score less or equal to 2 (Hewetson and Tallon 2021), and RDC wall thickness was less than 5 mm at ultrasound examination (Jones et al. 2003). Horses were housed in individual stalls and fed a commercial feed that was divided into twice daily feedings, which were consistent with the horses’ usual feeding regimen. Non-medicated water and straw were available ad libitum.

### Experimental design

**Fig. 1:**
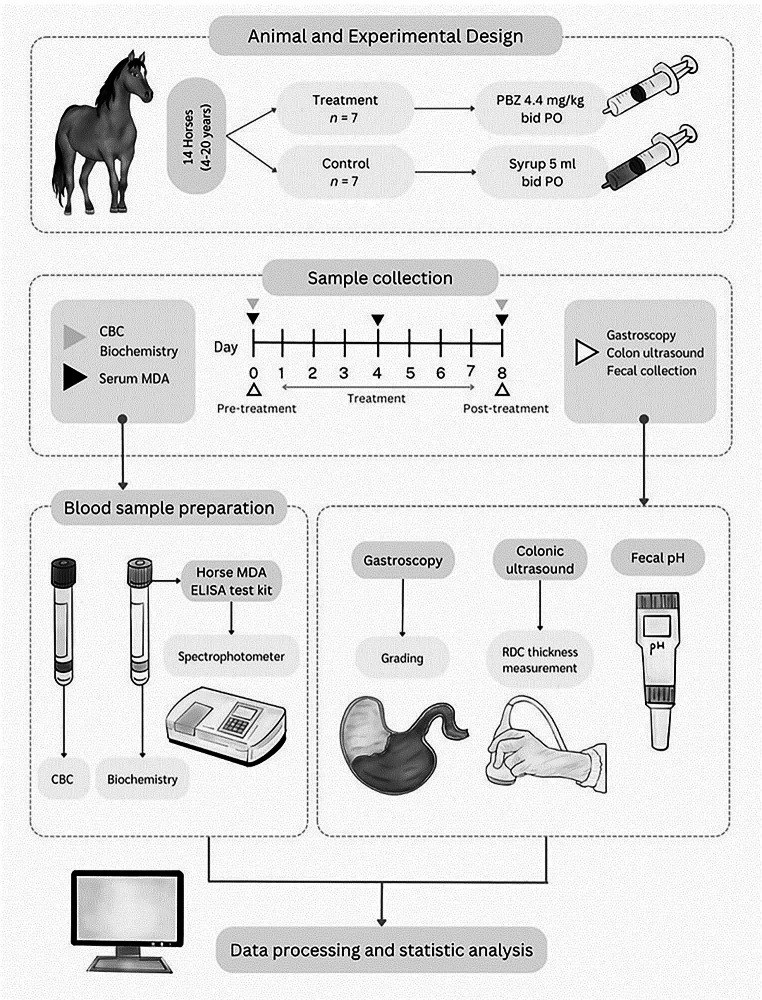
Graphic outline of the experimental study design

The experimental design is shown in Fig. 1. Horses were randomly assigned to the control (*n* = 7) or treatment (*n* = 7) group. Before the experiment, baseline data were collected, including gastrointestinal examinations by gastroscopy and ultrasonography, blood profiles (CBC and serum biochemistry), and fecal pH. During the investigation, the horses in the treatment group were administered syrup containing 4.4 mg/kg body weight (BW) of PBZ (Butalone Granules, Dechra, Australia) orally twice daily for 7 days (Nieto et al. 2012). The horses in the control group received syrup orally twice daily for 7 days as a placebo. General clinical parameters such as vital signs, behavior, water and food intake, attitude, defecation, and urination frequency were monitored throughout the study period. Blood samples were collected on Days 0, 4, and 8, while gastrointestinal examination and fecal sampling were performed on Days 0 and 8. Sampling time points were defined according to previous studies with similar experimental designs (Zuluaga Cabrera et al. 2016).

### Blood collection and analysis

Blood was collected from the jugular vein of each horse using a 20G, 1.5 inch needle. The amount of blood collected depended on the variables required for analysis. Three milliliters of blood per horse per time were collected in EDTA tubes for CBC, whereas 7 mL of blood was collected and transferred to plain tubes for serum biochemistry profiles. An automated blood analyzer was used to determine CBC (including red blood cell count (RBC), hematocrit (Hct), erythrocyte indexes, white blood cell count (WBC), differential WBC count, platelet count (PLT), platelet smear, and fibrinogen concentrations) and biochemistry profile (including total protein, albumin, creatinine concentrations) on Days 0 and 8. To assess of MDA concentrations, 7 mL of blood was collected from each horse on Days 0, 4, and 8. Serum was then separated by centrifugation and stored at − 20 °C until assayed.

### Gastroscopy

The presence of gastric lesions was investigated by gastroscopic examination, performed according to previously reported protocols (Andrew et al. 1999; Murray et al. 2001). Before gastroscopy, the horses were fasted for 15–18 h and water was withheld for 3–5 h (Murray 1994). The horses were restrained using stocks to ensure safety and minimize movement during the procedure. Sedation was induced by intravenous administration of 0.04 mg/kg BW acepromazine (Combistress, Kela N.V., Belgium) and 0.01 mg/kg BW detomidine (Detomo Vet, Ceva, Australia) (Barletta et al. 2023; Clark-Price and Mama 2022). To protect the gastroscope and prevent pharyngeal retroflexion, a nasogastric tube was introduced from the nostril through the stomach. A flexible video gastroscope with a 10.4 mm sheath diameter and 300 cm working length (PAL:60,130 PKSK, Karl Storz SE & Co., Germany) was inserted through a nasogastric tube for gastroscopic examination. Visualization was performed using the RP100 model with an endoscope (Karl Storz SE & Co.). During gastroscopy of each horse, a video was recorded for further grading. Five experienced veterinarians performed blind grading of the gastroscopic videos. Grading of EGUS, including both ESGD and EGGD, was performed according to previously reported guidelines (Banse and Andrews 2019; Sykes et al. 2015). The five veterinarians independently assessed the gastric mucosa before and after PBZ administration, and their grading scores were recorded. The median gastroscopic grading score for each horse was used for statistical analysis.

### Colonic ultrasonography

All horses were placed into restraining stocks for preparation before ultrasonography. The hair at the right intercostal spaces from 11th to 15th was clipped in a window-like pattern, with an imaginary line drawn dorsally from the shoulder to the tuber coxae and ventrally from the point of the elbow to the patella. The skin in this area was thoroughly cleaned using diluted chlorhexidine in warm water and wiped with alcohol. A low-frequency (2–3 MHz) curvilinear transducer (Transducer C5-2, Philips Ultrasound, Inc., WA, USA) was used to perform the examination. The RDC wall was visualized deeper to the right liver lobe, and its thickness was measured and recorded as previously described (Jones et al. 2003; le Jeune and Whitcomb 2014). Two veterinarians blinded to the horse group performed double measurements of the RDC wall thickness of each horse. For statistical analysis, the mean of repeated measures from two veterinarians was used for each horse.

### MDA analyses

Serum MDA concentrations were analyzed using the Horse MDA enzyme-linked immunosorbent assay (ELISA) kit according to the manufacturer’s instructions (MBS282731, MyBioSource, San Diego, CA, USA). This assay quantifies serum MDA concentrations in horses using a two-site sandwich ELISA validated for equines. Briefly, an MDA-specific antibody was precoated onto a microplate. The microplate wells were filled with standards and serum samples. The plate was covered and incubated at 37 °C for 2 h. After incubation and washing, a biotin conjugate was added to each well, and the microplate was incubated at 37 °C for 1 h. After the second incubation and washing, streptavidin–horseradish peroxidase was added to each well, and the microplate was incubated at 37 °C for 1 h. After the third incubation and washing, tetramethylbenzidine was added, and the microplate was incubated before adding sulfuric acid as a stopping solution. The degree of enzymatic turnover of the substrate was determined using a microplate reader set to 450 nm. A set of MDA calibrators was used to plot the calibration curve of absorbance versus MDA concentration. The serum MDA concentration of each sample was calculated from this standard curve. The assay detection range was 3.12−200.00 ng/mL, and the intra- and inter-assay coefficients of variability were approximately 8% and 12%, respectively.

### Fecal pH

Fecal samples were obtained directly from the rectum of each horse. In total, 50 g of fecal matter was promptly mixed with 50 mL deionized water. The resulting mixture was vortexed for 30 s, followed by a stabilization period of 5 min. Fecal pH levels were measured using a portable pH meter (Digicon pH-204, Germany) calibrated using pH 4 (potassium phthalate buffer) and pH 7 (potassium phthalate–monobasic sodium hydroxide buffer) for accuracy.

### Statistical analysis

Data were analyzed using IBM SPSS version 18.0 for Windows (NY, USA). The normal distribution of residuals from the statistical models was evaluated using the Shapiro–Wilk test. The results are reported as mean ± standard deviation (SD) for the blood profiles (except PLT, albumin, and fibrinogen concentrations) and fecal pH. The blood profiles (PLT, albumin, and fibrinogen concentrations) are reported as median ± Standard Error of the Mean (SEM). The results of gastric ulcer scoring, RDC wall thickness, and serum MDA concentration are expressed as median and interquartile ranges. Comparisons of blood profiles (except PLT, albumin, and fibrinogen concentrations) and fecal pH between the control and treatment groups were performed using the independent *t*-test. The paired *t*-test was used to analyze the time of evaluation (Days 0 and 8) within each group. The non-normally distributed data on blood profiles (PLT, albumin, and fibrinogen concentrations), gastric ulcer scoring, RDC wall thickness, and serum MDA concentrations were analyzed using the Mann–Whitney U test to compare the control and treatment groups at each time point. The Wilcoxon signed-rank test was used to analyze the results of blood profiles, gastric ulcer scoring, and RDC wall thickness at each time point (Days 0 and 8) within each group. The Friedman test was used to assess the MDA results at each time point (Days 0, 4, and 8) within each group. A *p* < 0.05 were considered statistically significant.

## Results

### General clinical parameters

Vital signs, water consumption, food intake, defecation, and urination remained normal in all horses throughout the experimental period.

### Blood profiles

The results of the hematological and biochemical analyses for the treatment and control groups are summarized in Table 1. Blood profiles of all horses remained within reference ranges. On Day 8, albumin concentrations in the treatment group were significantly lower than those in the control group (*p* = 0.001). In the treatment group, albumin (*p* = 0.001) and total protein concentrations (*p* = 0.001) were significantly lower on Day 8 than on Day 0.

**Table 1:** Hematology and biochemistry results

Parameters (units)	Control group			Treatment group			*P*-value between groups (Day 0)	*P*-value between groups (Day 8)	Reference Values
	Day 0	Day 8	*p*-value*	Day 0	Day 8	*p*-value**			
**WBC (10** ^**3**^ **/µL)**	7.05 ± 1.07	7.34 ± 0.80	0.49	8.39 ± 1.65	7.40 ± 1.96	0.10	0.10	0.94	5.60–12.10
**RBC (10** ^**6**^ **/µL)**	7.10 ± 0.81	7.13 ± 1.13	0.95	6.66 ± 0.79	6.47 ± 0.62	0.24	0.32	0.20	6.00–10.40
**Hct (%)**	35.31 ± 3.96	34.73 ± 3.90	0.78	33.10 ± 4.44	34.07 ± 7.78	0.74	0.34	0.85	27.00–45.00
**PLT (10** ^**3**^ **/µL)**	132.00 ± 16.34	132.00 ± 11.43	0.79	144.00 ± 20.11	135.00 ± 9.62	0.98	0.20	0.08	117.00–256.00
**Fibrinogen (g/dL)**	0.20 ± 0.08	0.20 ± 0.12	0.60	0.40 ± 0.09	0.40 ± 0.07	0.41	0.18	0.84	0.10–0.50
**Albumin (g/dL)**	3.20 ± 0.03	3.20 ± 0.04^A^	0.41	3.00 ± 0.10^a^	2.70 ± 0.06^b, B^	0.001	0.08	0.001	2.60–4.10
**Creatinine (mg/dL)**	1.96 ± 0.22	1.76 ± 0.23	0.52	1.96 ± 0.11	1.84 ± 0.26	0.19	0.87	0.67	0.40–2.20
**Total protein (g/dL)**	6.36 ± 0.46	6.27 ± 0.50	0.41	6.61 ± 0.33^a^	5.96 ± 0.48^b^	0.001	0.26	0.26	5.60–7.60

*WBC* White blood cell count, *RBC* Red blood cell count, *Hct* Hematocrit, *PLT* Platelet count

*P-value when comparing between Day 0 and Day 8 of control group

**P-value when comparing between Day 0 and Day 8 of treatment group

^a,b^The significant change was shown as *p* < 0.05 when comparing the values of Day 0 and Day 8

^A,B^The significant change was shown as *p* < 0.05 when comparing the values of control and treatment groups

### Gastroscopic findings

**Fig. 2:**
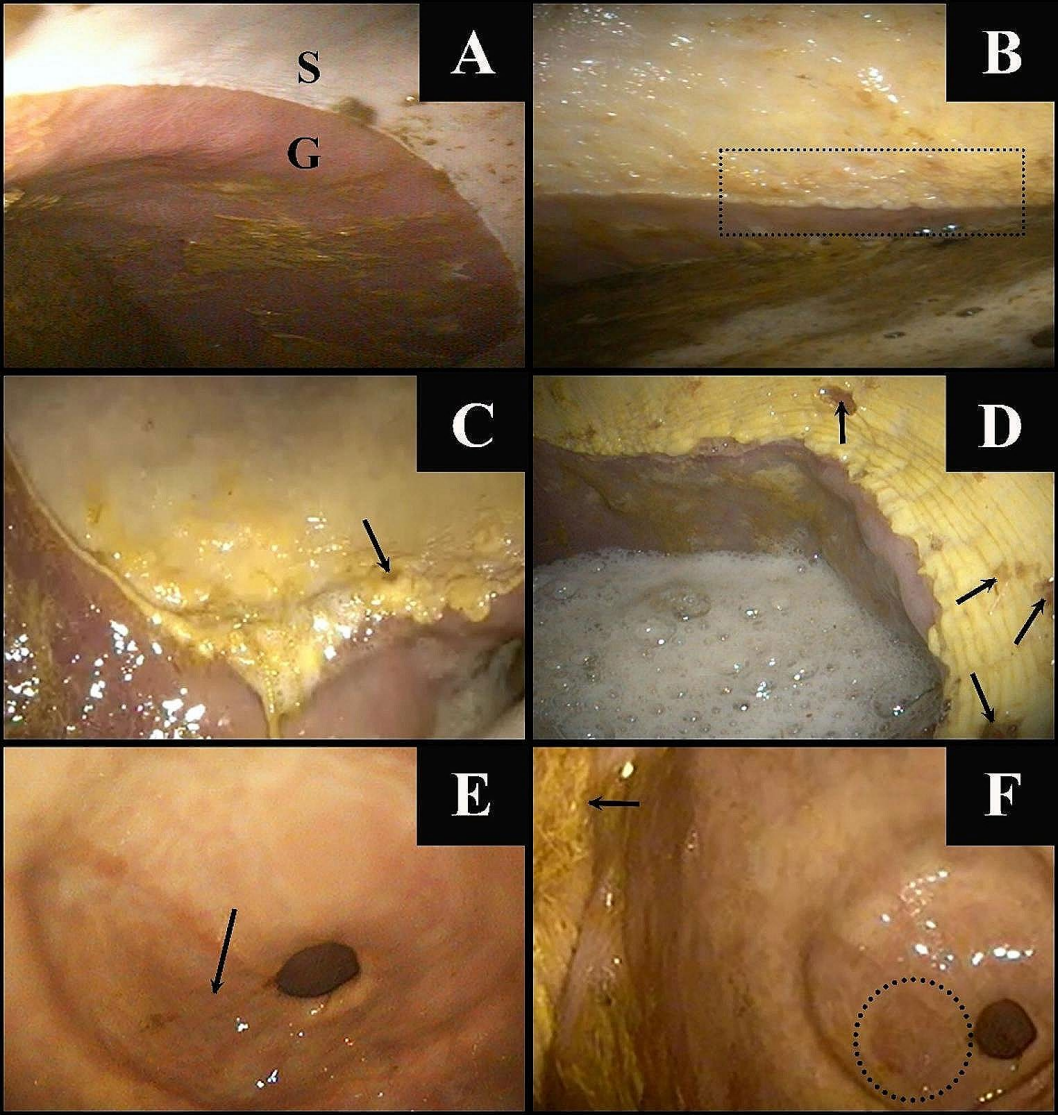
Examples of gastroscopic findings. **A** Grade 0: The epithelium exhibits intact structure, and no signs of hyperkeratosis are observed in either squamous (S) or glandular (G) parts. **B** Grade 1 squamous gastric disease: The mucosa shows hyperkeratosis (dotted line frame). **C** Grade 2 squamous gastric disease: The mucosa shows hyperkeratosis; a single ulcer lesion (arrow) is observed; the margo plicatus exhibits a rough and thickened appearance. **D** Grade 3 squamous gastric disease: Multifocal ulceration lesions (arrows) are observed around the margo plicatus. **E** Grade 1 glandular gastric disease: Areas of hyperemia (arrow) are observed in the pyloric region. **F** Grade 2 glandular gastric disease: The pyloric region appears diffusely hyperemic (dotted line frame) and focal fibrinosuppurative inflammation (arrow) is also present

**Fig. 3:**
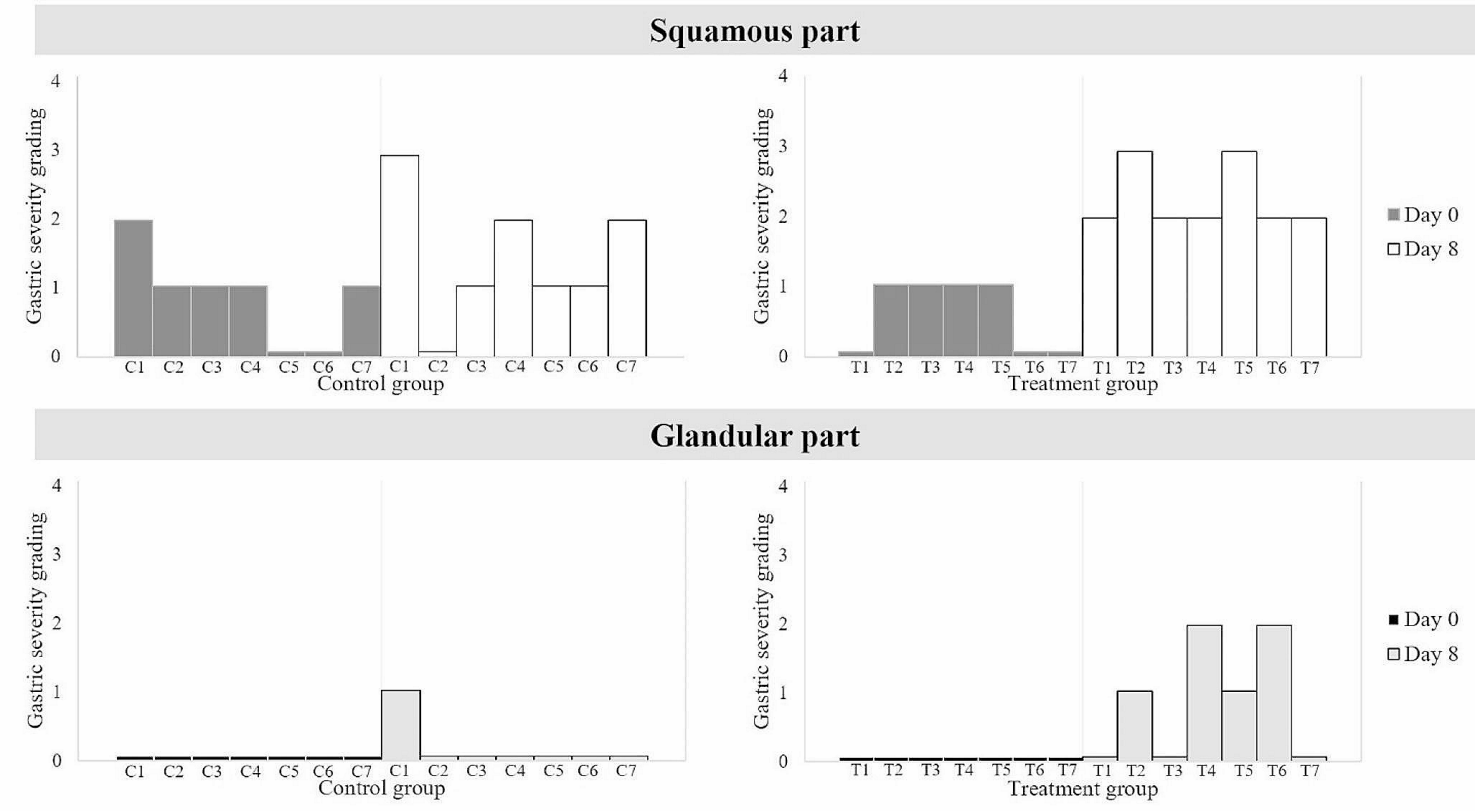
Gastric ulcer scores in all horses. The gastric ulcer of each horse is represented by a single bar in each group, colored by the day of evaluation (squamous part; dark gray for Day 0 and white for Day 8, glandular part; black for Day 0 and light gray for Day 8)

**Fig. 4:**
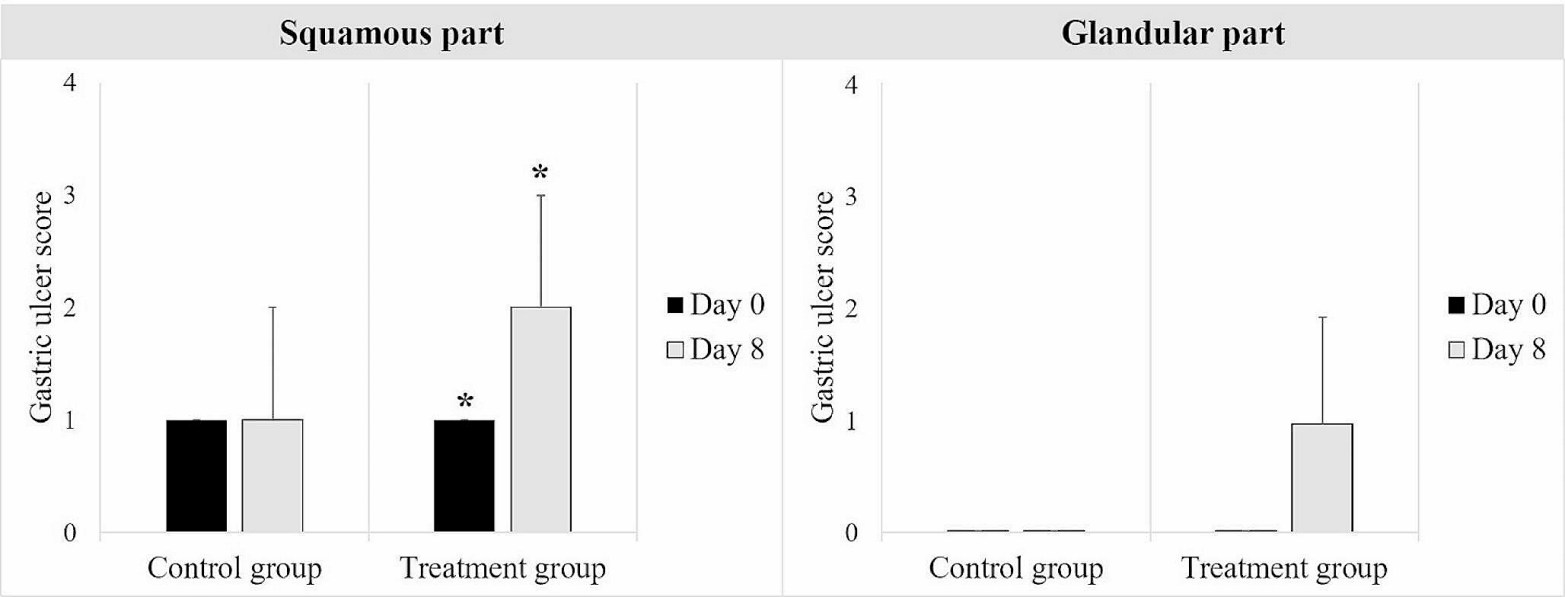
Comparison of median score of gastric ulcers between groups and time points in each group (black column Day 0; gray column Day 8). ^*^ a significant difference between the evaluation days within the same group (*p* = 0.01). Error bars represent the interquartile ranges

In each group and for each time point, the presence of gastric ulcers was graded separately for the squamous and glandular parts (Fig. 2). The gastric ulcer scores for all horses are shown in Fig. 3. In the treatment group, all horses had higher squamous gastric ulcer scores on Day 8 than on Day 0, whereas four out of seven horses had higher glandular gastric ulcer scores on Day 8 than on Day 0. In the control group, five out of seven horses had higher squamous gastric ulcer scores on Day 8 than on Day 0, whereas only one horse had higher glandular gastric ulcer scores on Day 8 than on Day 0. Comparisons of gastric ulcer scoring between groups are shown in Fig. 4. No significant difference was found between Days 0 and 8 for squamous or glandular ulcer in the control groups. Moreover, no significant difference was observed between the groups for either squamous or glandular ulcer at each time point. In the treatment group, the scores for squamous gastric ulcers were higher on Day 8 than on Day 0 (*p* = 0.01). In addition, a trend toward higher scores for glandular gastric ulcers was observed more on Day 8 compared to Day 0 (*p* = 0.06).

### Ultrasonographic findings

**Fig. 5:**
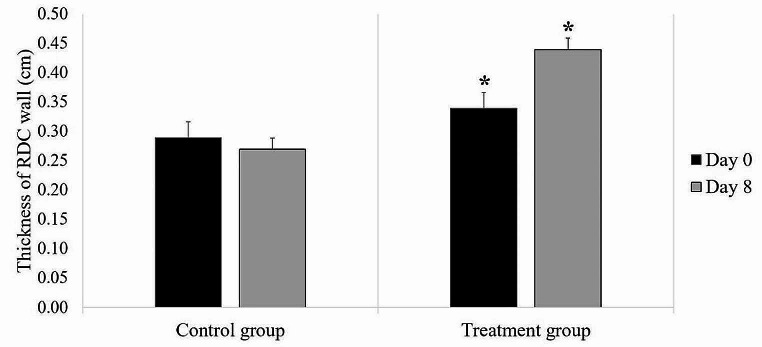
Comparison of the median thickness of the RDC wall between groups and time points in each group (black column Day 0; gray column Day 8). ^*^ a significant difference between the evaluation days within the same group (*p* = 0.03). Error bars represent the interquartile ranges

Comparisons of the RDC wall thickness between the groups and time points are shown in Fig. 5. In the treatment group, the RDC wall thickness on Day 8 (0.44 cm, range: 0.40 to 0.44 cm) was higher than that on Day 0 (0.34 cm, range: 0.29 to 0.36 cm; *p* = 0.03). No difference in RDC wall thickness was observed between each time point in the control group (Day 0: 0.29 cm, range: 0.25 to 0.34 cm; Day 8: 0.27 cm, range: 0.24 to 0.39 cm; *p* = 0.73). No difference in RDC wall thickness was noted between the groups on Day 0 (control group: 0.29 cm, range: 0.25 to 0.34 cm; treatment group: 0.34 cm, range: 0.29 to 0.36 cm; *p* = 0.48) or Day 8 (control group: 0.27 cm, range: 0.24 to 0.39 cm; treatment group: 0.44, range: 0.40 to 0.44 cm; *p* = 0.07).

### Serum MDA concentrations

**Fig. 6:**
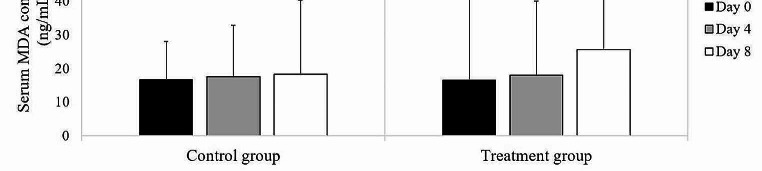
Comparison of the median serum MDA concentrations between groups and time points in each group (black column Day 0; gray column Day 4; white column Day 8). Error bars represent the interquartile ranges

The MDA concentrations were compared between the groups and over time (Fig. 6). No differences in MDA concentrations were found between the evaluation time points of the control group (Day 0: 16.71 ng/mL, range: 8.39 to 27.97 ng/mL; Day 4: 17.56 ng/mL, range: 3.58 to 32.77 ng/mL; Day 8: 18.33 ng/mL, range: 5.97 to 40.38 ng/mL; *p* = 0.32) and the treatment group (Day 0: 16.47 ng/mL, range: 8.75 to 41.61 ng/mL; Day 4: 17.92 ng/mL, range: 6.79 to 40.06 ng/mL; Day 8: 25.68 ng/mL, range: 8.89 to 61.70 ng/mL; *p* = 0.12). No differences in MDA concentrations were observed between the study groups on Day 0 (*p* = 0.78), Day 4 (*p* = 0.75), and Day 8 (*p* = 0.13).

### Fecal pH

**Fig. 7:**
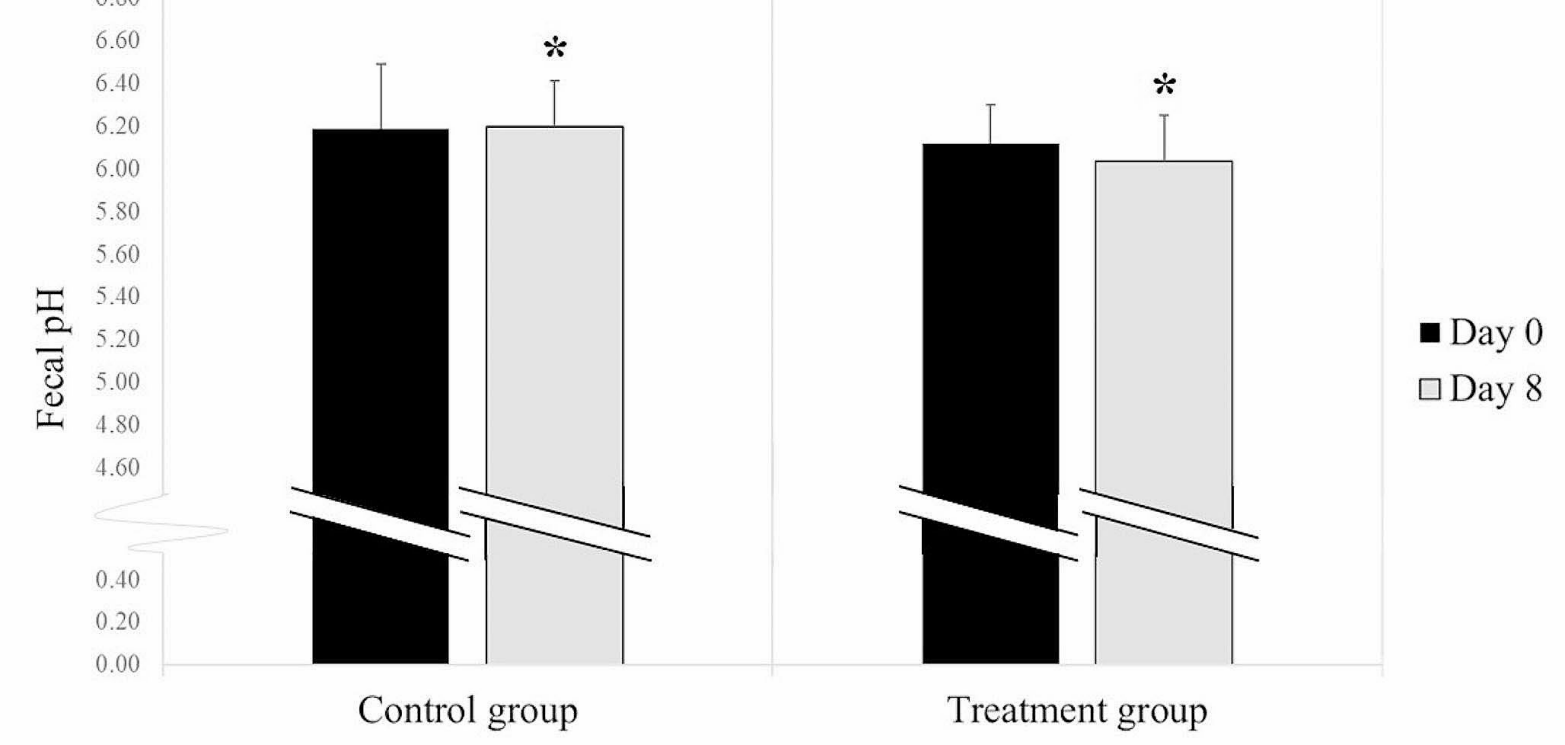
Comparison of the mean fecal pH between groups and time points in each group (black column Day 0; gray column Day 8). ^*^ a significant difference between groups within the same evaluation day (*p* = 0.001)

Comparisons of fecal pH between the groups and time points are reported in Fig. 7. On Day 8, the fecal pH in the treatment group (6.04 ± 0.22) was lower than that in the control group (6.20 ± 0.18) (*p* = 0.001). No difference in fecal pH was observed between the evaluation time points of the control group (Day 0: 6.19 ± 0.30; Day 8: 6.20 ± 0.18; *p* = 0.74) and treatment group (Day 0: 6.12 ± 0.22; Day 8: 6.04 ± 0.22; *p* = 0.46).

## Discussion

This study assessed the hematological effects, gastrointestinal tract changes, and serum MDA concentrations in horses after oral PBZ administration at clinical doses. Our findings were consistent with those of a previous study that used the same dose of PBZ administration showing a significant decrease in the average albumin and total protein concentrations in the PBZ treatment group (MacAllister et al. 1993). The association between hypoproteinemia and gastrointestinal ulceration in horses remains controversial. A previous study reported microvascular injury from PBZ administration associated with forming duodenal and colonic erosions (Meschter et al. 1990). Moreover, administering an oral PBZ dose of 4.4 mg/kg every 12 h for 14 days, as indicated by an earlier study, led to lowered total protein and albumin concentrations. This suggests that the decrease in total protein and albumin levels may be markers of intestinal permeability and protein loss subsequent to PBZ administration (Ricord et al. 2021). The present study assumed that the decline in these variables may be attributed to enteropathy following PBZ administration. Although the various hematologic parameters in the PBZ treatment group in the current study remained within the reference range, prolonged administration of PBZ may induce significant alterations in blood profiles, potentially affecting the overall health of the horses.

The results of the present study are consistent with those of previous studies with regard to the upward trend of glandular gastric ulcer scores observed after PBZ administration (MacAllister et al. 1993; MacKay et al. 1983; Monreal et al. 2004). NSAIDs are known disruptors and inhibitors of COXs, which are responsible for converting arachidonic acid into prostaglandin E2 (PGE2) (Burke et al. 2010). In animals, PGE2 and PGI2 play key roles in suppressing gastric acid secretion. Earlier studies found that COX inhibition by PBZ decreased the levels of PGs and augmented acid secretion, thereby lowering the gastric pH (Engevik et al. 2020; Tobin et al. 1986). This inhibition compromises PG-mediated vasodilation and regulates histamine-induced acid secretion, rendering the gastrointestinal mucosa susceptible to acid invasion and subsequent necrosis and ulceration (Fitzpatrick 2004; MacAllister 1983). Moreover, PGs are critical for maintaining epithelial tight junction integrity and are essential for mucosal repair, barrier function, and overall mucosal health of the equine gastrointestinal system (Sanchez 2018). Furthermore, PGs contribute to epithelial restitution and mucosal cell turnover (Zushi et al. 1996). In the present study, the majority of horses exhibited increased glandular gastric ulcer scores after receiving PBZ. However, this increase was not statistically significant, which aligns with findings from a previous study indicating that administering PBZ at a dose of 4.4 mg/kg twice daily did not reduce PGE2 levels (Pedersen et al. 2018). Additionally, it is hypothesized that PBZ administration at clinical doses might affect gastrointestinal PG function differently from PBZ overdose, which is known to induce EGGD (Sanchez 2018; Zushi et al. 1996). Our findings showed significant gastric squamous mucosa changes after PBZ administration. The present study proposed that the synergistic effect of PBZ and other factors may contribute to ESGD. A recent study found that factors that prolong the exposure of the squamous mucosa to acid can intensify ESGD (Hewetson and Tallon 2021). Moreover, feeding straw is associated with a higher prevalence of ESGD because of its rough texture and high lignin content (Luthersson et al. 2009). Our treatment group was fed straw as roughage combined with PBZ administration, which may have lowered gastric pH and damaged the squamous mucosa. Moreover, the horses in these studies received reduced amounts of grass or hay because of limited forage availability in the paddock, potentially leading to insufficient acid buffering in the squamous mucosa (Argenzio 1999). Notably, in the present study, 5 of 7 horses in the control group showed increased squamous gastric scores after placebo administration. We assumed that the increased scores are attributed to pre-sedation fasting. Although the fasting duration was relatively short, it may have induced a rapid decrease in gastric pH because horses continuously produce gastric acid over 12–24 h. Moreover, fasting reduces saliva production, sodium bicarbonate, and the ability to buffer gastric hydrochloric acid (Andrews et al. 2006), potentially predisposing horses to gastric ulcers. In addition, the endoscopic gastroscopy technique would have induced additional stress because of the prolonged fasting and water deprivation required before the procedure (Murray 1994), thereby contributing to the high gastric ulcer scores. The short interval between the two fasting periods in this study (7 days) may also have had an impact, potentially contributing to the increased gastric ulcer scores. Gastroscopy is currently the primary antemortem method for definitive ulcer confirmation. Typically, an endoscope measuring at least 3 m is used to visualize both the squamous and glandular gastric sections (Andrews et al. 2002). Endoscopic gastroscopy may predispose horses to stress because it requires prolonged fasting and water deprivation before the procedure (Murray 1994). Moreover, the equipment is expensive, difficult to use in the field, and impractical for a large population. Given these limitations, other methods need to be explored for the early diagnosis of gastric ulcers, especially those that are simple, cost-effective, painless, safe, and noninvasive. For instance, the use of serum biomarkers is an appealing screening strategy.

The present study found a significant increase in the wall thickness of the RDC on ultrasonography after PBZ treatment, which is consistent with the findings of a previous study (Jones et al. 2003). A study found that 13 horses with a history of NSAID exposure, particularly PBZ, exhibited multifocal to coalescing ulcerative foci exclusively in the RDC. Colonic wall hyperplasia was evident histologically, characterized by thickened bands of fibrous connective tissues and fibrinonecrotic debris (Karcher et al. 1990). These lesions may appear in horses within a week after receiving a high dose of NSAIDs. Right dorsal colitis is caused by various factors. PBZ can increase the risk of inflammation in the RDC by reducing anion secretion, particularly HCO_3_^-^ and Cl^-^ (Bauck et al. 2022; Vinijkumthorn et al. 2024). Dehydration can reduce fluid volumes in the digestive tract, thereby worsening NSAID toxicity when administered to dehydrated horses (Vinijkumthorn et al. 2024). The present study postulated that the horses evaluated may have been susceptible to dehydration due to their prolonged exposure to the paddock throughout the day, especially considering that this study was performed during the summer season. Although the clinical dose of PBZ administered in this study impacted the RDC, the RDC wall thickness remained within the normal range of less than 5 mm (Galvin et al. 2004); thus, the potential side effect of this drug may become evident if used over an extended period. Moreover, right dorsal colitis, which increases the wall thickness of the RDC, induces changes in mucosal integrity. Changes in the integrity of the intestinal mucosa due to inflammation can cause protein-losing enteropathy. Consequently, hypoproteinemia and hypoalbuminemia are found as adverse effects of NSAIDs (Reed et al. 2006).

Fecal pH measurement is a noninvasive method for evaluating the health of the equine hindgut. A previous study showed that a neutral fecal pH is optimal in horses, whereas a fecal pH within the range of 6.0 − 6.2 is considered indicative of subclinical hindgut acidosis (Richards et al. 2006). Our study revealed that the fecal pH in the PBZ treatment group was significantly lower than that in the control group. This finding is consistent with that of an earlier study that suggested a relationship between stress and the development of more severe squamous gastric ulcer scores in young horses engaged in crib-biting. The horses that engaged in crib-biting had a noticeable decrease in fecal pH compared with those that did not (Nicol et al. 2002). To the best of our knowledge, no studies have investigated the impact of oral NSAID administration on fecal pH in horses. Our study found that administering PBZ medication is associated with lower fecal pH. Prolonged and extensive use of this drug poses a potential risk to horses because a sustained decrease in fecal pH could lead to adverse outcomes. This concern arises from the understanding that maintaining proper hindgut pH is crucial for microbial balance, and a pH below 6.0 may contribute to problems, such as overgrowth of gram-positive bacteria, loss of gram-negative bacteria, production of endotoxins, and manifestation of clinical conditions, such as diarrhea, colic, and laminitis (Garner et al. 1978).

MDA is a byproduct of cell membrane lipid peroxidation that serves as a biomarker of oxidative stress in various biological samples (El-Deeb et al. 2018). It has also been proposed as a biomarker for NSAID-induced lesion development in the gastric mucosa of rats and humans (Naito et al. 1998). Moreover, MDA has been evaluated as a biomarker of intestinal ischemia–reperfusion injury in cats, rats, and dogs (Lantz et al. 1992; Nosalova et al. 2007). Our study showed increased serum MDA concentrations after treatment, although the differences with the baseline were not significant. A previous study that induced gastric ulcers by administering PBZ orally and prolonging the fasting period reported increased MDA concentrations in both gastric juice and blood, implying that PBZ administration during fasting led to the generation of free radicals that caused local and systemic effects in horses (Zuluaga Cabrera et al. 2016). Similarly, another study on horses with a treatment history of injectable PBZ for chronic musculoskeletal pain conditions revealed a notable increase in MDA concentrations in non-survivors (El-Ashker et al. 2012). Moreover, in a rat study on gastric mucosal injury induced by analgesic-antipyretic drugs, elevated in MDA concentrations were noted in the gastric mucosa following the administration of diclofenac, indicating oxidative damage (Sanchez et al. 2002). In our opinion, the absence of statistical significance may be attributed to the high variability in serum MDA concentrations, potentially reducing statistical power. Furthermore, MDA is classified as a nonspecific biomarker for diagnosing gastric ulcers in horses (Shawaf et al. 2020), which can also respond to oxidative stress induced by other causes, such as muscle damage from physical exercise (Chiaradia et al. 1998). This study assumed that PBZ administration at clinical doses may induce oxidative stress, but did not yet induce statistically significant changes in serum MDA concentrations at the time of measurement. Treatment duration is another factor that may be relevant, as longer treatment durations could potentially lead to a significant increase. However, this factor may not be beneficial for utilizing the marker as an early detection tool. Consequently, further studies should focus on identifying candidate proteins that can serve as early indicators of damage to the equine gastrointestinal tract arising from PBZ treatment. Because of the flexibility in selecting PBZ therapeutic regimens for horses at the recommended levels, further investigations should explore the effects of a shorter duration of PBZ administration or of different dosages.

## Conclusion

This study initially explored the hematological impact and changes in the gastrointestinal tract while evaluating serum MDA concentrations in horses administered clinical doses of PBZ (4.4 mg/kg BW twice daily for 7 days). Consistently with previous studies, this study revealed hematological and gastrointestinal changes, including decreased plasma proteins and albumin, increased squamous gastric ulcer score, and thickening of the RDC wall. Additionally, decreased fecal pH was observed. These alterations occurred in the absence of obvious clinical signs. Serum MDA concentrations increased after PBZ administration, even though statistical differences were not observed. Our findings highlight the need for veterinarians to closely monitor the gastrointestinal condition of horses receiving PBZ treatment, even at clinical doses. However, this study indicates that MDA may not be the most appropriate early detection biomarker for gastrointestinal damage caused by PBZ treatment in horses. Further studies should focus on identifying specific protein biomarkers for the early detection of gastrointestinal damage in horses after prolonged PBZ treatment, which would provide a crucial diagnostic tool to promptly address potential life-threatening health problems associated with PBZ use.

## Data Availability

No datasets were generated or analysed during the current study.
